# A knowledge-guided strategy for improving the accuracy of scoring functions in binding affinity prediction

**DOI:** 10.1186/1471-2105-11-193

**Published:** 2010-04-17

**Authors:** Tiejun Cheng, Zhihai Liu, Renxiao Wang

**Affiliations:** 1 State Key Laboratory of Bioorganic Chemistry, Shanghai Institute of Organic Chemistry, Chinese Academy of Sciences, 345 Lingling Road, Shanghai 200032, PR China

## Abstract

**Background:**

Current scoring functions are not very successful in protein-ligand binding affinity prediction albeit their popularity in structure-based drug designs. Here, we propose a general knowledge-guided scoring (KGS) strategy to tackle this problem. Our KGS strategy computes the binding constant of a given protein-ligand complex based on the known binding constant of an appropriate reference complex. A good training set that includes a sufficient number of protein-ligand complexes with known binding data needs to be supplied for finding the reference complex. The reference complex is required to share a similar pattern of key protein-ligand interactions to that of the complex of interest. Thus, some uncertain factors in protein-ligand binding may cancel out, resulting in a more accurate prediction of absolute binding constants.

**Results:**

In our study, an automatic algorithm was developed for summarizing key protein-ligand interactions as a pharmacophore model and identifying the reference complex with a maximal similarity to the query complex. Our KGS strategy was evaluated in combination with two scoring functions (X-Score and PLP) on three test sets, containing 112 HIV protease complexes, 44 carbonic anhydrase complexes, and 73 trypsin complexes, respectively. Our results obtained on crystal structures as well as computer-generated docking poses indicated that application of the KGS strategy produced more accurate predictions especially when X-Score or PLP alone did not perform well.

**Conclusions:**

Compared to other targeted scoring functions, our KGS strategy does not require any re-parameterization or modification on current scoring methods, and its application is not tied to certain systems. The effectiveness of our KGS strategy is in theory proportional to the ever-increasing knowledge of experimental protein-ligand binding data. Our KGS strategy may serve as a more practical remedy for current scoring functions to improve their accuracy in binding affinity prediction.

## Background

Molecular recognition plays an important role in many fundamental processes in biological systems [1,2]. The basic concept of molecular recognition was first narrated by Emil Fischer more than 100 years ago. His "lock-and-key" theory [3], i.e. "... *enzyme and glycoside must fit together like a key and a lock in order to initiate a chemical action upon each other*...", has long been regarded as the basis for studying the binding between a ligand molecule to its biological receptor. Molecular docking, as a computational simulation of the ligand-receptor binding process, is widely applied in many research areas, such as structure-based drug design. For example, docking-based virtual screening [4-7] has become a complementary approach to high-throughput screening for the discovery of novel lead compounds, which is popular among academic research groups as well as pharmaceutical companies. In such a process, a library of small molecules are fit into the binding pocket of a given target protein through molecular docking, aiming at achieving an optimal complementarity of steric and physicochemical properties. Then, a computational method, which is often referred to as "scoring function", is used to evaluate the fitness between the ligand and the protein. All of the molecules are subsequently ranked by their binding scores, and only the most promising ones will be examined later in experiments. Obviously, a quantitative prediction of protein-ligand binding affinities is the key to the success of such studies.

As demonstrated in many previous studies [8,9], today's molecular docking programs, such as DOCK [10], AutoDock [11-13], FlexX [14], Surflex [15,16], LigandFit [17], GOLD [18,19], and Glide [20,21], are able to identify the correct binding pose of a flexible ligand to its receptor with a reasonable accuracy. However, binding affinity prediction (the "scoring problem") is still the Achilles' heel of molecular docking because in many cases the binding scores produced by scoring functions do not correlate well with true binding affinities, sometimes even cannot rank a set of compounds correctly [22-27]. The relatively disappointing performance of scoring functions in this aspect may be the result of a compromise between accuracy and speed since simplifications have to be made regarding solvation effects, conformational flexibility, and other factors in protein-ligand binding. A number of computationally more expensive methods have also been developed for binding affinity prediction in the past two decades or so. These methods typically conduct conformational sampling of the protein-ligand complex of interest through extensive molecular dynamics simulation in explicit solvent. Thus, they are able to address solvation effect and conformational flexibility in theory. Free energy pathway methods, such as free energy perturbation [28] and thermodynamics integration [29], sometimes can reproduce protein-ligand binding affinities within 1~2 kcal/mol. Nevertheless, they are normally used for the computation of the relative binding affinities of closely resembled ligand molecules, and thus have rather limited applications. "End-point" methods, such as the linear interaction energy (LIE) approximation [30,31] and the MM-PB/SA method [32], avoid the integration of free energy pathway in order to save computation cost. They are normally applied to the modeling of a congeneric set of compounds binding to the same target protein. A number of successful applications of these methods have already been reported in literature. However, their success seems to rely on well-selected systems, and their robustness still needs to be validated more extensively. Inaccuracy in force field or inadequate sampling may account for their possible failures. In fact, some comparative tests [33] indicate that such methods are not necessarily more accurate than scoring functions although they definitely consume more computational resources. Technically, these methods are by far too computationally expensive for high-throughput tasks. It is also complicated to set up a job with such methods. Due to these concerns, such methods are not likely to be integrated into molecular docking programs for practical uses.

Considering the balance between accuracy, efficiency, and applicability, scoring functions are still the best choice to tackle the scoring problem for molecular docking and some other tasks in structure-based drug design. Thus, improving the general performance of scoring functions is undoubtedly a worthwhile aim. A good number of scoring functions have already been reported in literature since 1990s. They can be classified roughly into three categories: (i) Force field-based methods [10-13,18,19] rely on established force fields to compute the non-covalent interactions between protein and ligand, including van der Waals and electrostatic interactions. They are often augmented by GB/SA or PB/SA terms in order to consider solvation effect. (ii) Empirical scoring functions [20,21,34-43] decompose the protein-ligand binding free energy into some basic terms, such as hydrogen bonding, hydrophobic effect and so on. Each term is computed with an intuitive algorithm, and the weight factors of each term are typically derived from a regression analysis on a set of protein-ligand complexes with known binding affinities. Hence, empirical scoring functions are also referred to as regression-based methods. (iii) Methods based on potentials of mean force [44-52] compute protein-ligand interactions as a sum of distance-dependent pairwise potentials. A technical advantage of these methods is that deduction of potentials of mean force only requires the knowledge of protein-ligand complex structures.

Most of today's scoring functions are developed as all-purpose models, which are presumably applicable to all sorts of protein-ligand complexes. A recent comparative assessment of 16 popular scoring functions conducted by us [22] revealed that an accurate prediction of the binding affinities across a variety of protein-ligand complexes is still a major challenge for them. Nevertheless, it was also noticed that on certain classes of complexes, some scoring functions actually produced very promising results. This observation indicates that it is perhaps more practical to develop specific scoring functions applicable to certain classes of protein-ligand complexes in order to improve the accuracy in binding affinity prediction. In fact, this idea has been practiced by some researchers. For instance, Teramoto et al. introduced the supervised scoring modes as well as an optimized consensus scoring scheme with feature selection to enhance the enrichment factor in structure-based virtual screening [53,54]. Recently, Seifert and co-workers published a review article on target-specific scoring functions (or "targeted scoring functions") [55]. Current all-purpose scoring functions can be re-calibrated on certain classes of protein-ligand complexes to become target-specific, which is probably the most straightforward approach. For example, the DrugScore-RNA [56] adopts the same framework as DrugScore [45,46], a scoring function based on potentials of mean force, but is derived from 670 crystal structures of nucleic acid-ligand and nucleic acid-protein complexes. Antes proposed the POEM approach (Parameter Optimization using Ensemble Methods) [57] and applied it to the optimization of two scoring functions (FlexX and ScreenScore) on kinases and ATPases. Seifert described a statistical method (ProPose) for improving the signal-to-noise ratio of scoring functions in molecular docking and successfully customized the Böhm scoring function on three selected proteins: cyclin dependent kinase 2, estrogen receptor, and cyclooxygenase 2 [58].

The targeted scoring functions mentioned above typically require re-parameterization or special customization on current scoring functions to become suitable for specific systems. In this study, we have proposed an alternative strategy, namely knowledge-guided scoring (KGS). This strategy requires no re-parameterization in prior, and in principle can be applied in combination with any scoring functions to any classes of protein-ligand complexes. The key idea is that the unknown binding affinity of a given protein-ligand complex can be estimated more reliably based on the known binding affinity of an appropriate reference complex. The reference complex is required to share a similar pattern of key protein-ligand interactions with the given protein-ligand complex. For this purpose, our KGS strategy utilizes a sufficient number of relevant protein-ligand complexes with known structures and binding affinities as a knowledge set. The key protein-ligand interactions in each complex are summarized as a pharmacophore model, which can be elucidated by an automatic algorithm implemented by us. The knowledge set is then searched through for the appropriate reference complex for any given protein-ligand complex. Thus, our KGS strategy can take full advantage of known knowledge, resulting in an improved accuracy in binding affinity prediction. In our study, the KGS strategy was tested in combination with two all-purpose scoring functions, i.e. X-Score [35] and PLP [37,38] on three sets of protein-ligand complexes. An improved average accuracy for both X-Score and PLP was indeed obtained. Detailed descriptions are given in the following sections.

## Methods

### Overall strategy

Our basic assumption is that molecular systems with similar structures have similar properties, a strategy that has been applied successfully to the computation of some physicochemical properties such as partition coefficient [59] and water solubility [60]. Accordingly, the unknown binding affinity of a given complex can be estimated more reliably from the known binding affinity of a reference complex, which shares a similar pattern of protein-ligand interactions with the query complex. The binding scores provided by a reasonable scoring function should correlate well with experimentally determined binding data as follows:(1)

Here,  denotes for the expected binding affinity of a reference protein-ligand complex (*R*); *R*_*score*, *SF *_denotes for the binding score of this complex calculated by a scoring function *SF*; while *b *and *k*, respectively, are the intercept and the slope of the regression line between the binding scores and experimentally measured binding data of a set of protein-ligand complexes. Similarly, the expected binding affinity of a query protein-ligand complex (*Q*) calculated by the same scoring function is:(2)

By subtracting Equation 1 from Equation 2, one has:(3)

Replacing the expected binding affinity of *R *with the known experimental value (*R*_*exp*_), one has:(4)

Equation 4 indicates how the binding affinity of a given protein-ligand complex is computed using the known binding affinity of a proper reference complex as a starting point. For the convenience of narration, this scoring strategy will be referred to as the KGS strategy, i.e. Knowledge-Guided Scoring, throughout this article. In principle, any scoring method may be employed to compute the required binding scores of both the reference complex and the query complex in Equation 4. Nevertheless, it is certainly more reasonable in reality to choose a capable scoring method for this purpose. The reference complex can be selected among a database of protein-ligand complexes with reliable structures and binding data. The constant *k *in Equation 4 can be derived through a regression analysis between the experimental binding data and the computed binding scores by the employed scoring method on the same database. It is introduced to scale the outcomes of scoring functions, which could be in arbitrary units, to a realistic range comparable to the experimental binding data of the reference complex.

### Pharmacophore elucidation

**Figure 1:**
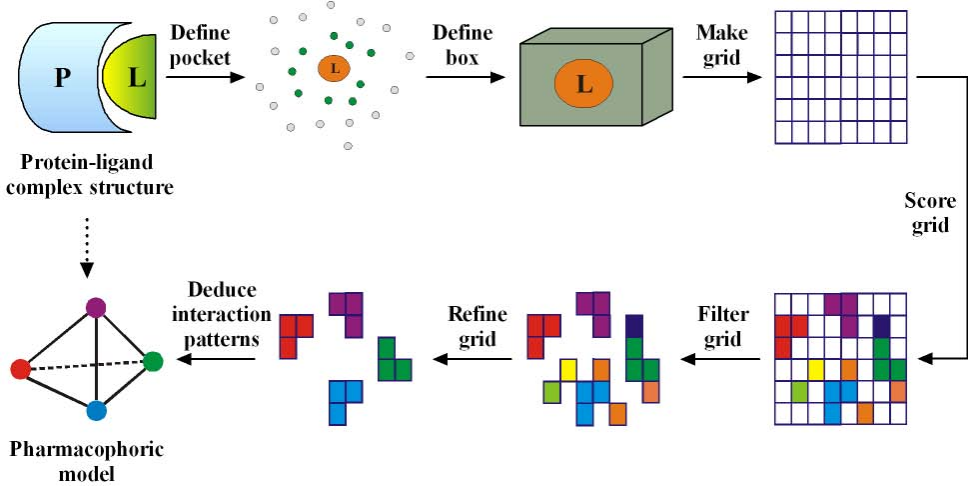
**Illustration of our algorithm for pharmacophore elucidation**.

**Figure 2:**
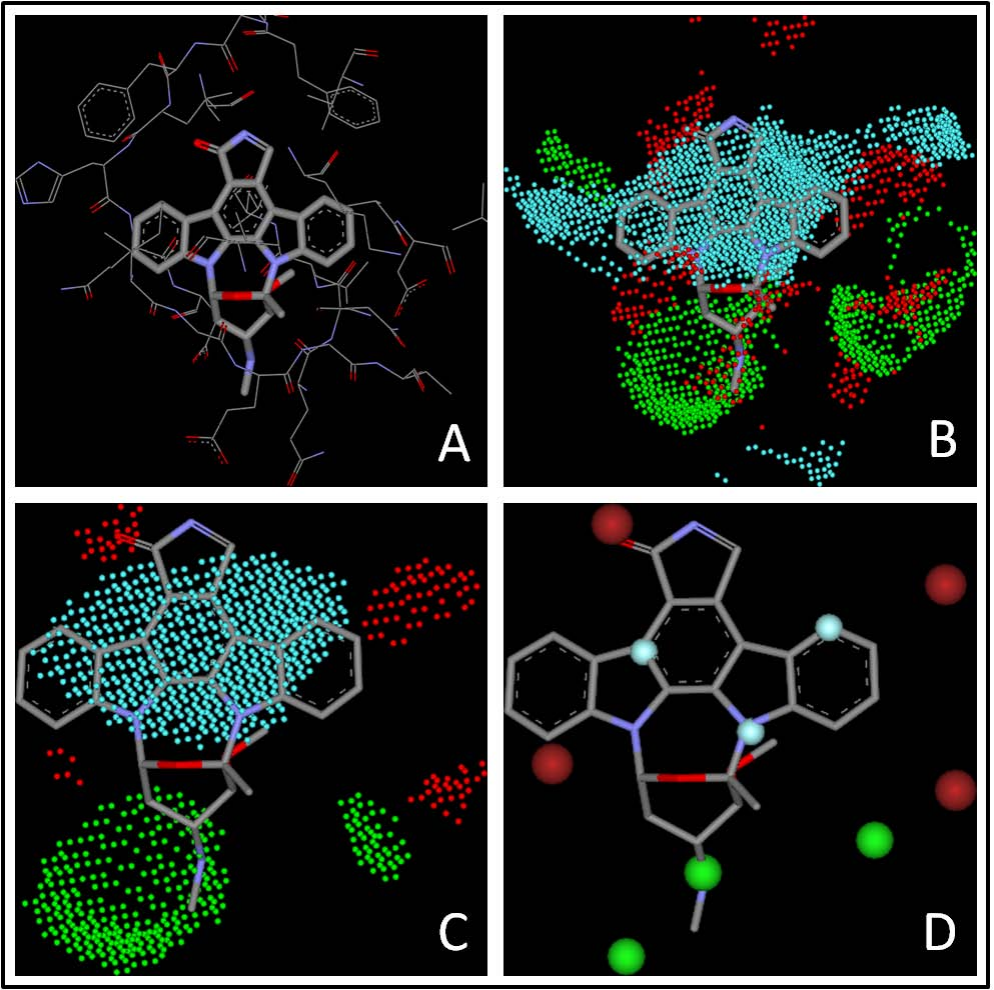
**The pharmacophore mode of the CDK2-staurosporine complex (PDB entry **1AQ1**) elucidated by our algorithm**. **(A) **Binding mode of staurosporine to CDK2. **(B) **The grids with significant contributions to binding. **(C) **Grids after refinement. **(D) **The final pharmacophore model. In each figure, features of hydrogen bond donor, hydrogen bond acceptor, and hydrophobic center are represented by dots/balls in green, red, and cyan, respectively.

Considering the complicated nature of a protein-ligand binding process, choosing the proper reference complexes is obviously critical for the success of Equation 4. If complexes *Q *and *R *share a similar pattern of protein-ligand interactions, our basic assumption of "similar structures have similar properties" is more likely to be true. Thus, the core algorithm of our KGS strategy is how to define the right reference complex. In our study, the pattern of key protein-ligand interactions is presented as a pharmacophore model, which is actually the three-dimensional arrangement of a set of features including hydrogen bond donor, hydrogen bond acceptor, and hydrophobic center. Our algorithm for receptor-based pharmacophore elucidation is similar to the one used by the Pocket module implemented in the LigBuilder software [61,62]. The overall flowchart of our algorithm is shown in Figure 1. The results produced by this algorithm on a CDK2-inhibitor complex (PDB entry 1AQ1) are illustrated in Figure 2 as an example.

The only input needed by our algorithm is the three-dimensional structure of a given protein-ligand complex, which can be an experimentally resolved structure or a computer model generated by molecular docking or other methods. In either case, the ligand molecule is required to be inside the binding site on the protein with the desired binding pose. The very first step of our algorithm is to identify the amino acid residues on the protein which are in direct contact with the bound ligand molecule. An amino acid residue is considered to be in direct contact with the ligand if any heavy atom on it is within a distance of 5.0 Å from any heavy atom on the ligand. This distance cutoff is adjustable to the users. A box large enough is then used to enclose all of these residues as well as the ligand molecule (Figure 1). Next, evenly spaced grids are created inside the box with a spacing of 0.5 Å by default. Each grid is checked for its accessibility by placing a hydrogen atom on it. If the hydrogen probe bumps with any protein atom, i.e. when the inter-atom distance of the two participating atoms is shorter than the sum of their van der Waals radii minus 0.5 Å, the grid under consideration will be labeled as "ignored". The van der Waals atomic radii used in our algorithm are cited from the Tripos force field. If a grid is more than 5.0 Å away from any atom on the protein, it will also be labeled as "ignored", as there will be no direct interaction between the protein and any ligand atom placed on this grid. All "ignored" grids will be removed later on to speed up the following processes. As result, the remaining grids actually define the binding pocket for subsequent analyses.

For each remaining grid, three different types of probes are placed on it and the binding score between each probe and the protein are evaluated thereby. These probes include (1) a positively charged sp^3 ^nitrogen atom (ammonium cation), representing a hydrogen bond donor; (2) a negatively charged sp^2 ^oxygen atom (as in a carboxyl group), representing a hydrogen bond acceptor; and (3) a sp^3 ^carbon atom (methane), representing a hydrophobic group. The binding scores between each probe and the protein are calculated by using the corresponding algorithms in the empirical scoring function X-Score (version 1.2) [35]. Final classification of each grid will be determined according to the particular probe which produces the highest binding score, either as "donor", "acceptor", or "hydrophobic". For example, a grid labeled as "donor" indicates that a hydrogen bond donor is mostly preferred on this particular grid. All of the scored grids are further refined and clustered by a two-step process. At the first step, the average score is calculated over all donor grids. The donor grids whose scores are lower than the average score are re-labeled as "ignored". The same process is also repeated on the acceptor grids and the hydrophobic grids. Consequently, only the grids with significant contributions to protein-ligand interactions will survive (Figure 1 & Figure 2B). At the second step, our algorithm checks each remaining donor grid and counts the total number of its "neighbors", i.e. the remaining grids of the same type within a range of 2.0 Å. The average number of neighbors for all donor grids is calculated. Those grids with a total number of neighbors below the average will be re-labeled as "ignored" and filtered out. The same process is also repeated on all acceptor grids and the hydrophobic grids. After this process, only the grids in aggregation will still survive, which represent the key interaction sites inside the binding pocket more clearly (Figure 1 & Figure 2C).

Finally, a pharmacophore model is deduced based on the outcomes of all previous steps. A pharmacophore feature is used to represent each group of grids of the same type (see Figure 2D). The center of each pharmacophore feature locates on the grid with the highest "pharmacophore score" (*PS*), which is computed as follows:(5)

Here, *PS*_*i *_is the pharmacophore score of the *i *th grid under consideration, *S*_*j *_is the binding score of the *j *th neighboring grid of this grid, and *r*_*ij *_is the distance between the *i *th grid and the *j *th grid. According to this algorithm, the pharmacophore score of a certain grid combines the contributions from all its neighboring grids within 2.0 Å. The spatial distribution of the neighboring grids also has an impact: a dense group of grids will be associated with a higher pharmacophore score; whereas the pharmacophore score for a sparse group of grids will be somewhat lower. Besides, a correction is introduced in Equation 5 to favor the pharmacophore features overlapping with the atoms on the ligand: The *d*_*i *_in Equation 5 is the distance between the *i *th grid and a close non-hydrogen atom on the given ligand. Thus, if a pharmacophore feature overlaps exactly with a certain atom on the ligand, its pharmacophore score will retain by 100%; otherwise its pharmacophore score will receive a certain distance-dependent discount. In order to avoid the generation of too many pharmacophore features, our algorithm by default sets the minimal distance between two features to 3.5 Å, approximately the average van der Waals distance between two non-hydrogen atoms. The above process is repeated until all groups of grids are attributed to certain pharmacophore features. Occasionally, too many pharmacophore features are deduced by our algorithm when the binding pocket is really large. Thus, an upper limit of 15 features in a pharmacophore model is set for the sake of subsequent similarity searching. By using our program, the average computation time consumed by pharmacophore elucidation for a typical protein-ligand complex (with ~30 residues at the binding site) was merely 170 ms on a low-end laptop with one 2.13 GHz CPU and 2GB memory inside.

### Pharmacophore mapping

**Figure 3:**
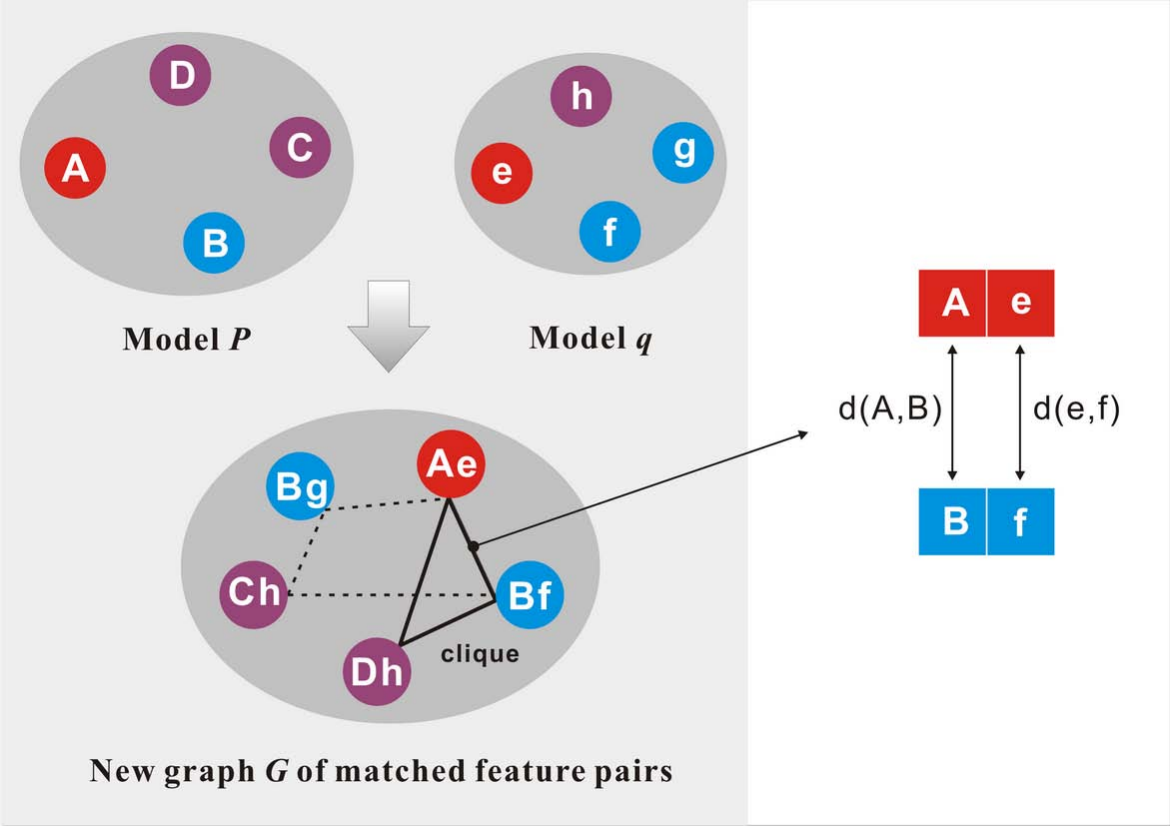
**Illustration of the algorithm for finding the common features between two pharmacophore models *P *and *q***.

As implied above, the key interactions in the formation of a protein-ligand complex is characterized by a pharmacophore model. By our KGS strategy, the reference complex for a given query complex is the one sharing the maximal similarity in this aspect with the query complex. Since a pharmacophore model is basically a set of vertices in space, the problem of defining the similarity between two pharmacophore models turns out to be equivalent to finding the common vertices between two sets of vertices. An algorithm was implemented in our program for this purpose (Figure 3). Firstly, all possible matched pairs of features between the pharmacophore models *P *and *q *are identified. Two features of the same type are considered to match each other. For example, feature *A *in model *P *matches feature *e *in model *q*. Secondly, a hypothetical graph *G *is constructed on-the-fly using each matched pair of features as a new node. Two nodes, e.g. *Ae *and *Bf*, are connected with an edge if the *A-B *distance in model *P *is close enough to the *e-f *distance in model *q*. Here, two distances *d*_1 _and *d*_2 _are considered to be close to each other if *d*_1 _<k *d_2 _(when *d*_1 _>*d*_2_) or *d*_2 _<k *d_1 _(when *d*_1 _<*d*_2_). *k *is an adjustable parameter with a default value of 1.1. Then, the Born-Kerbosch [63] clique detection algorithm is applied to identify the maximal clique in graph *G*. The maximal number of common features between models *P *and *q *in turn equals to the number of the nodes in the maximal clique. Consequently, the similarity index (*SI*) between models *P *and *q *is calculated by the Tanimoto method [64]:(6)

Here, *N*_*P *_and *N*_*q *_are the numbers of pharmacophore features in models *P *and *q*, respectively; while *N*_*Pq *_is the maximal number of common features found between *P *and *q*.

For any given protein-ligand complex, an external database of known protein-ligand complexes supplied by the user will be examined with the algorithm described above. The complex with the highest similarity index, if higher than a user-set cutoff, is chosen as the reference in Equation 4 for calculating the binding affinity of the query complex. By using our program, the similarity search is fairly fast, which took less than 1 ms to map a pair of pharmacophore models on a low-end laptop with one 2.13 GHz CPU and 2GB memory inside.

### Preparation of test sets

Three sets of protein-ligand complexes selected from the PDBbind database [65,66] were used as the test sets in our study. The PDBbind database provides a collection of experimentally determined binding data of the protein-ligand complexes deposited in the Protein Data Bank (PDB) [67]. The protein-ligand complexes considered in this study were all selected from the "refined set" of the PDBbind database (version 2007), which consists of 1300 protein-ligand complexes with high-quality structures and reliable binding data. Compilation of the PDBbind refined set has been described in one of our recent publications [22]. Briefly, each qualified protein-ligand complex in this data set has the following features:

(1) The complex structure is resolved through X-ray crystal diffraction with an overall resolution better or equal to 2.5 angstroms. Neither the protein nor the ligand has any missing fragment in the crystal structure.

(2) Binding data of the complex is experimentally measured as either dissociation constant (*K*_*d*_) or inhibition constant (*K*_*i*_). Both the protein and the ligand used in binding assay match exactly the ones used in structure determination.

(3) The complex is formed by one protein molecule and one ligand molecule in a binary manner, and the binding is non-covalent in nature. The ligand molecule does not contain any uncommon elements, such as Be, B, Si and metal atoms, and its molecular weight does not exceed 1000.

Each test set was composed of a number of protein-ligand complexes formed by one particular type of protein, including 112 HIV protease complexes, 44 carbonic anhydrase complexes, and 73 trypsin complexes, respectively (see Table 1). These three proteins were chosen since they were the three most populated ones in the PDBbind refined set. The structural files of all of these complexes were downloaded from PDB. They were then processed so that they could be readily utilized by the modeling software available to us. Firstly, a complete biological unit of each complex was split into a protein molecule and a ligand molecule. Atomic types and bond types of the ligand molecule were automatically assigned by the I-interpret program [68]. They were then visually inspected and corrected if necessary. Hydrogen atoms were added to the protein and the ligand by using the SYBYL [69] software. For the sake of convenience, both the protein and the ligand were set according to a simple protonation scheme under neutral pH: all carboxylic acid and phosphonate groups were deprotonated; while all aliphatic amine and guanidino/amidino groups were protonated. All water molecules included in the crystal structure were removed. Metal ions, if residing inside the binding pocket and forming coordinate bonds to the ligand (such as in the case of carbonic anhydrase), were kept with the protein molecule. No structural optimization was performed on either the protein or the ligand in order to retain their coordinates exactly the same as those in the original PDB file.

**Table 1:** PDB codes of the protein-ligand complexes in the three test sets

**HIV protease complexes (*N *= 112)**
1GNM, 1GNN, 1GNO, 1A30, 1A9M, 1AAQ, 1AJV, 1AJX, 1B6J, 1B6K, 1B6L, 1B6M, 1BDQ, 1BV7, 1BV9, 1BWA, 1BWB, 1C70, 1D4K, 1D4L, 1D4Y, 1DIF, 1DMP, 1G2K, 1G35, 1HBV, 1HEG, 1HIH, 1HII, 1HOS, 1HPO, 1HPS, 1HPV, 1HPX, 1HSH, 1HVH, 1HVI, 1HVJ, 1HVK, 1HVL, 1HVR, 1HVS, 1HWR, 1HXB, 1HXW, 1IIQ, 1IZH, 1IZI, 1LZQ, 1MES, 1MET, 1MEU, 1MRW, 1MRX, 1MSM, 1MSN, 1MTR, 1NH0, 1ODY, 1OHR, 1PRO, 1QBR, 1QBS, 1QBT, 1QBU, 1SBG, 1SDT, 1SDU, 1SDV, 1SGU, 1SH9, 1T7J, 1W5V, 1W5W, 1W5X, 1W5Y, 1Z1H, 1Z1R, 1ZP8, 1ZPA, 1ZSF, 1ZSR, 2AOC, 2AOD, 2AOE, 2AQU, 2AVM, 2AVO, 2AVQ, 2AVS, 2AVV, 2BPV, 2BPY, 2BQV, 2F80, 2F81, 2F8G, 2FGU, 2FGV, 2HB3, 2I0A, 2I0D, 7HVP, 7UPJ, 2HS2, 2AOG, 2HS1, 1A94, 1AID, 1KZK, 1TCX, 3AID
**Trypsin complexes (*N *= 73)**
1C1R, 1C5P, 1C5Q, 1C5S, 1C5T, 1CE5, 1F0T, 1F0U, 1K1I, 1K1J, 1K1L, 1K1M, 1K1N, 1OSS, 1PPC, 1PPH, 1QB1, 1QB6, 1QB9, 1QBN, 1QBO, 1TNG, 1TNH, 1TNI, 1TNJ, 1TNK, 1TNL, 1V2J, 1V2K, 1V2L, 1V2N, 1V2Q, 1V2R, 1V2S, 1V2T, 1V2U, 1V2W, 2A31, 2BZA, 2FX6, 1BRA, 1G3B, 1G3C, 1G3D, 1G3E, 1GHZ, 1GI1, 1GI4, 1GI6, 1GJ6, 1J16, 1J17, 1O2H, 1O2J, 1O2K, 1O2N, 1O2O, 1O2Q, 1O2S, 1O2W, 1O2X, 1O2Z, 1O30, 1O33, 1O36, 1O38, 1O3D, 1O3F, 1O3H, 1O3I, 1O3J, 1O3K, 1V2O
**Carbonic anhydrase complexes (*N *= 44)**
1BN1, 1BN3, 1BN4, 1BNN, 1BNQ, 1BNT, 1BNU, 1BNV, 1BNW, 1A42, 1AVN, 1BCD, 1CIL, 1CIM, 1CIN, 1CNW, 1CNX, 1CNY, 1G1D, 1G45, 1G46, 1G48, 1G4J, 1G4O, 1G52, 1G53, 1G54, 1I9L, 1I9M, 1I9N, 1I9O, 1I9P, 1I9Q, 1IF7, 1IF8, 1OKL, 1TTM, 1XPZ, 1XQ0, 1YDA, 1YDB, 1YDD, 2EZ7, 2H4N

In addition, a set of putative binding poses were prepared for the ligand molecule in each complex in all three test sets. These binding poses were generated with the GOLD software (version 4.1) by docking the native ligand into its co-crystallized protein target. The parameter "No. of GA operations" was set to 10000 to get docking poses as diverse as possible. The ChemScore scoring function implemented in GOLD was chosen as the scoring engine. All other parameters were assigned the default values. A total of 100 top-ranked docking poses were retained for each ligand. The root-mean-square deviations (RMSD) from the native ligand pose of each docking pose was calculated using the "*rms_analysis*" utility in GOLD.

Regarding the HIV protease test set, at least one docking pose with RMSD < 2 Å from the native binding pose are found among all GOLD-generated docking poses for 90 ligands out of the total 112. It indicates that near-native poses were successfully generated for most ligands with our method. The average RMSD of the docking poses for these 90 ligands was 5.2 Å, indicating that those docking poses were also diverse. Another 19 ligands had at least one docking pose with RMSD falling in the range of 2~3 Å. Our method failed to produce docking poses with RMSD < 3 Å only for three ligands in the HIV protease test set. These three cases were all associated with large and flexible ligand molecules. As for the carbonic anhydrase and the trypsin test sets, 39 out of 44 and 64 out of 73 ligands, respectively, had at least one docking pose with RMSD < 2 Å from the native ligand pose among all GOLD-generated docking poses. Finally, the corresponding native binding pose observed in crystal structure was added to the ensemble of GOLD-generated docking poses for each ligand to ensure that the most important point in the conformational space of each ligand was sampled.

### Evaluation of scoring methods

Our recent study [22] revealed that two scoring functions, i.e. X-Score [35] and PLP [37,38], have relatively better performance in binding affinity prediction than other scoring functions. They were thus chosen in this study to test the KGS strategy. Note that X-Score has three built-in options, i.e. HPScore, HMScore, and HSScore. In many cases, the difference in the outcomes of these three options is marginal. For the sake of convenience, only the average value of these three options in X-Score was considered in our study. Similarly, PLP also has two variations, i.e. PLP1 and PLP2. Only PLP1 was considered in our study. The X-Score program was obtained from its original authors. PLP1 was implemented by us according to the descriptions given in the original references [37,38] as well as the information given in the user manual of the Discovery Studio software (version 2.0) [70]. We compared the results produced by our in-house implementation of PLP1 and those produced by the one implemented in Discovery Studio on the entire PDBbind refined set. These two sets of results were found to be almost identical (data not shown), indicating that our own implementation of PLP1 was correct.

For each test set, both X-Score and PLP1 were used to compute the binding scores of all member protein-ligand complexes. For each given complex, the pharmacophore mapping algorithm described in a previous section was applied to identify a proper reference complex among the other complexes included in the same test set. If such a reference complex was found, an adjusted binding score of the given protein-ligand complex was computed with Equation 4 based on the known binding constant of the reference complex. If not, the binding score of the given protein-ligand complex was unchanged. This process was repeated until all protein-ligand complexes in each test set had been processed. Then, the Pearson correlation coefficient (*R*_*p*_) between the experimentally determined binding constants and the final binding scores was calculated. The standard deviations (*SD*) in fitting the computed binding scores to the experimental binding constants were used as a quantitative measurement of the accuracy of each scoring method for comparison. The smaller is the standard deviation, the better is the accuracy.

The computations described above were all based on experimentally resolved protein-ligand complex structures, and the corresponding results are referred to as Set I throughout this article. In the reality of binding affinity prediction, however, one needs to rely on predicted structures in most cases. Thus, it is necessary to test our KGS strategy in such scenarios as well. For this purpose, a total number of 100 putative docking poses were prepared for each ligand in all three test sets. For each complex, all of these docking poses as well as the native binding pose were computed by X-Score and PLP, respectively, using the same procedure described in the previous paragraph. The binding score of each docking pose was adjusted using Equation 4 whenever applicable. Then, the best binding score obtained across all docking poses and the native binding pose for each protein-ligand complex was considered in the correlation analysis with the experimental data. In other words, the docking poses generated by the GOLD software were actually rescored by X-Score and PLP in combination with our KGS strategy. The corresponding results are referred to as Set II throughout this article.

## Results and Discussion

### Performance on HIV protease complexes

As revealed in our recent evaluation on scoring functions [22], virtually no scoring function was able to provide reasonable predictions of the binding affinities of HIV protease complexes. For example, the correlation coefficients (*R*) between the experimental binding constants of the 112 HIV protease complexes in our test set and the binding scores computed by X-Score and PLP are 0.329 and 0.190, respectively (Table 2). The disappointing performance of these two scoring functions may be attributed to the relatively large and flexible binding site of HIV protease, which remains as a challenge for scoring functions as well as other scoring methods.

**Table 2:** Statistical results produced by two scoring functions alone on the three test sets

	X-Score			PLP		
**Test set**	***R*^*a*^**	***SD*^*b*^**	***k*^*c*^**	***R*^*a*^**	***SD*^*b*^**	***k*^*c*^**

**HIV protease complexes** **(N = 112)**	0.329	1.55	0.664	0.190	1.61	-0.0099

**Carbonic anhydrase complexes** **(N = 44)**	0.648	1.06	2.045	0.690	1.01	-0.0833

**Trypsin complexes** **(N = 73)**	0.815	0.98	1.988	0.762	1.09	-0.0518

^*a *^Pearson correlation coefficient between the experimental binding constants of these complexes and the binding scores produced by the given scoring function.

^*b*^Standard deviation in regression (in log*K*_*d *_units)

^*c*^Slope of the regression line, which is needed in Equation 4.

**Figure 4:**
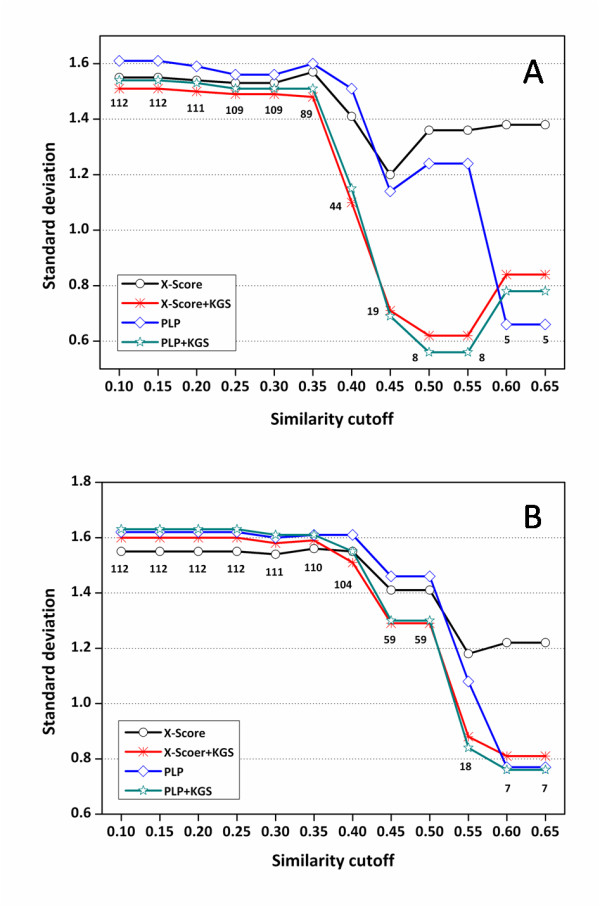
**Standard deviations (in log *K*_a _units) in fitting the experimentally measured binding constants of the HIV protease complexes and the binding scores computed by X-Score and PLP in combination with the KGS strategy**. (*A*) Results obtained based on crystal structures (Set I). (*B*) Results obtained based on docking poses (Set II). The X axis indicates the similarity cutoffs used in defining reference complexes. The numbers indicated on this figure are the total numbers of the complexes considered at each similarity cutoff.

The standard deviations produced by X-Score and PLP by using crystal structures are plotted as a function of the similarity cutoffs considered in reference searching in Figure 4A. Detailed statistical results can be found in the Additional file 1, Table S1. One can see that the standard deviations produced by X-Score+KGS, i.e. X-Score in combination with the KGS strategy, are consistently lower than those produced by X-Score alone, indicating that the KGS strategy is really helpful to improve the accuracy in binding affinity prediction. In particular, the improvement is obvious when the similarity cutoff used in defining the reference complex is above 0.40, and the standard deviations of X-Score+KGS drop below 1.00 log units. This is logical since our KGS strategy is not supposed to be effective if the reference complex does not share a similarity high enough to the query complex. The similarity cutoff of 0.40, by which KGS starts to work, actually can be expected. We noticed that a medium-size ligand molecule typically occupies four to five pharmacophore features in binding pocket (see Figure 2 for an example). Assuming that both the ligand molecule in the query complex and the ligand molecule in the reference complex occupy five pharmacophore features and three out of the five features match between the query and the reference, the similarity index would be 3/(5 + 5 - 3) = 0.43 according to Equation 6.

**Figure 5:**
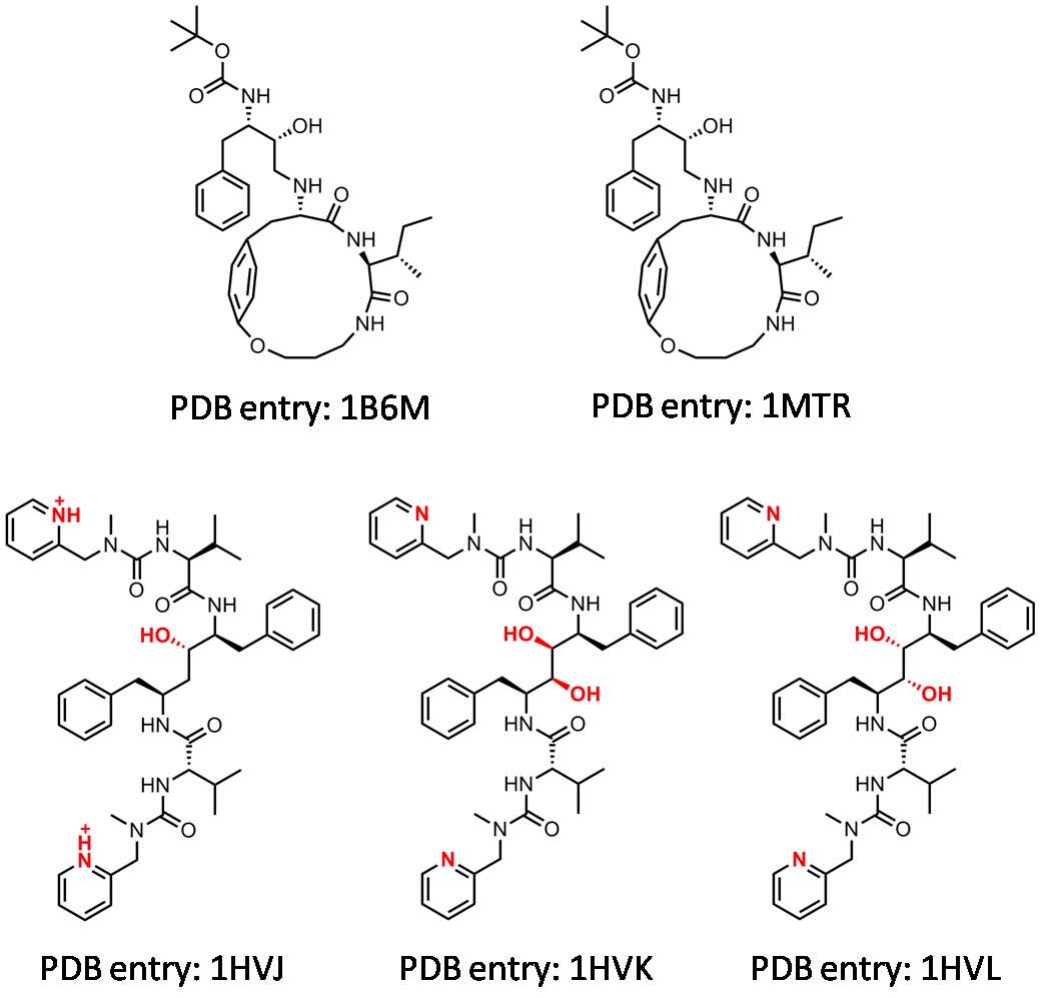
**Chemical structures of the five ligand molecules in the HIV protease complexes listed in Table 3**.

The five pairs of HIV protease complexes with the highest similarity identified by our algorithm are summarized in Table 3. The chemical structures of the five ligand molecules in the complexes are shown in Figure 5. One can see that the ligand in PDB entry 1B6M is actually identical to the one in its reference, i.e. PDB entry 1MTR; while the ligands in PDB entries 1HVJ, 1HVK, and 1HVL are basically stereoisomers to those in their references. These findings indicate that our algorithms for pharmacophore deduction and mapping are capable to identify complexes sharing similar patterns of protein-ligand interactions. Note that X-Score underestimated the absolute binding constants of complexes 1HVJ, 1HVK, and 1HVL consistently by 1-2.5 log units. After the application of our KGS strategy, this systematic deviation has been largely corrected, resulting in a much reduced average error.

**Table 3:** Information on the five pairs of HIV protease complexes with the highest similarities

The query complex				The reference complex		
**PDB** **Code**	**-log*K*_*d *_(exp)**	**X-Score+** **KGS**	**X-Score**	**PDB** **Code**	**-log*K*_*d*_(exp)**	**Similarity**

1B6M	8.40	8.39	8.83	1MTR	8.40	0.67
1MTR	8.40	8.41	8.84	1B6M	8.40	0.67
1HVJ	11.40	10.68	8.86	1HVK	10.96	0.67
1HVK	10.96	11.68	9.28	1HVJ	11.40	0.67
1HVL	9.95	11.43	8.90	1HVJ	11.40	0.67

The results produced by PLP in combination with the KGS strategy (Figure 4A) basically reveal the same trend: at lower similarity cutoffs, the improvements exhibited by PLP+KGS over PLP alone are not obvious; while at higher similarity cutoffs, considerable improvements are observed, demonstrating the success of the KGS strategy again. An exception is that PLP alone produced acceptable results on the five complexes listed in Table 3, but this can be well interpreted as a coincidence. Interestingly, considerable improvements are also observed above the similarity cutoff of 0.40, exactly the same as the case of X-Score+KGS. In addition, the corresponding results produced by X-Score+KGS and PLP+KGS are really close on all subsets of complexes when the similarity cutoff > 0.40 although the performance of two scoring functions alone can be very different. These findings further support our statement that the success of the KGS strategy is in principle independent from the scoring method employed in computation.

When computer-generated docking poses are considered in scoring for instead, i.e. Set II, the standard deviations produced by X-Score and PLP as a function of the cutoffs used in similarity search are plotted in Figure 4B. Detailed statistical results can be found in the Additional file 1, Table S2. The same trend as in Set I results are observed for both X-Score and PLP: the performance of both scoring functions is improved by the KGS strategy when the similarity cutoff used in reference searching is higher than 0.40. The standard deviations of both scoring functions are lowered by 0.2 units or even more. This observation indicates that our KGS strategy can be applied not only to the complexes with experimentally determined structures but also predicted structures, e.g. the docking poses generated by a molecular docking program. This feature may make KGS a valuable strategy for "real" drug design tasks, such as structure-based virtual screening. It should be mentioned that the standard deviations at different similarity cutoffs in Set II results are somewhat larger than the counterparts in Set I results. It is understandable since for some complexes in this test set, the native binding pose of the ligand molecule is not necessarily the best-scored binding pose selected by scoring function, which is a well-known phenomenon [22,26,27]. This defective aspect of scoring function of course introduces extra noises in binding affinity prediction.

### Performance on carbonic anhydrase complexes

**Figure 6:**
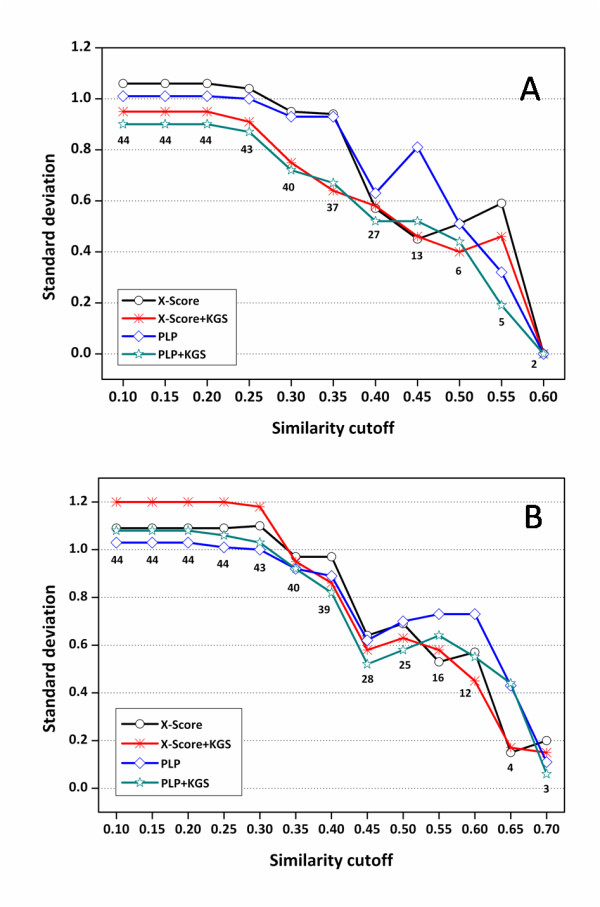
**Standard deviations (in log*K*_a _units) in fitting the experimentally measured binding constants of the carbonic anhydrase complexes and the binding scores computed by X-Score and PLP in combination with the KGS strategy**. (*A*) Results obtained based on crystal structures (Set I). (*B*) Results obtained based on docking poses (Set II). The X axis indicates the similarity cutoffs used in defining reference complexes. The numbers indicated on this figure are the total numbers of the complexes considered at each similarity cutoff.

Compared to the performance on the HIV protease test set, X-Score and PLP are more successful on this test set. The standard deviations calculated by these two scoring functions are 1.06 and 1.01 log units, respectively (Table 2). The standard deviations produced by X-Score, PLP and those in combination with the KGS strategy on this test set are plotted as a function of the similarity cutoffs used in reference searching in Figure 6. Detailed statistical results are summarized in the Additional file 1, Tables S3 and S4.

When only the crystal structures are considered in scoring (Set I), one can see that the standard deviations produced by X-Score+KGS at various similarity cutoffs are consistently lower than or comparable to those produced by X-Score alone (Figure 6A). The same trend retains when the scoring method is switched to PLP. When the docking poses are considered in scoring (Set II), the standard deviations produced by X-Score+KGS and PLP+KGS are basically lower than those produced by X-Score and PLP alone when the similarity cutoff is above 0.40 (Figure 6B). In both cases, however, the improvement after the application of the KGS strategy is not as significant as the one observed on the HIV protease test set. It is understandable since as mentioned above, the performance of X-Score and PLP alone is already good on this test set, leaving not much room for improvement. Considering the intrinsic accuracy of the employed scoring functions as well as the uncertainties in experimental binding data, there is certainly a limit on the average accuracy of binding affinity prediction. Note that after the similarity cutoff in defining the reference complexes is raised above 0.40, the standard deviations produced by X-Score+KGS and PLP+KGS are consistently below 0.60 log units (corresponding to ~0.8 kcal/mol in binding free energy at room temperature). We believe that this level of accuracy, if having not reached the limit of scoring functions, should be rather close to it.

Another trend observed in Figure 6 is that X-Score+KGS and PLP+KGS produced comparable statistical results especially when the similarity cutoffs are relatively high. In contrast, there is noticeable difference in the statistical results produced by X-Score and PLP alone under the same circumstances. In fact, exactly the same trend can be observed on the HIV protease test set as well (Figure 4). Each scoring function has its own strength and weakness, and thus one would expect that different scoring functions produce different results on the given systems. Therefore, the users have to test on their selected targets a number of scoring functions or even combinations of scoring functions before any prediction can be made. Our results indicate that once scoring functions are combined with the KGS strategy, they tend to produce converged results since the difference between their outcomes are largely leveled off by the use of a reference. This feature will bring great convenience to the users in practice.

### Performance on trypsin complexes

**Figure 7:**
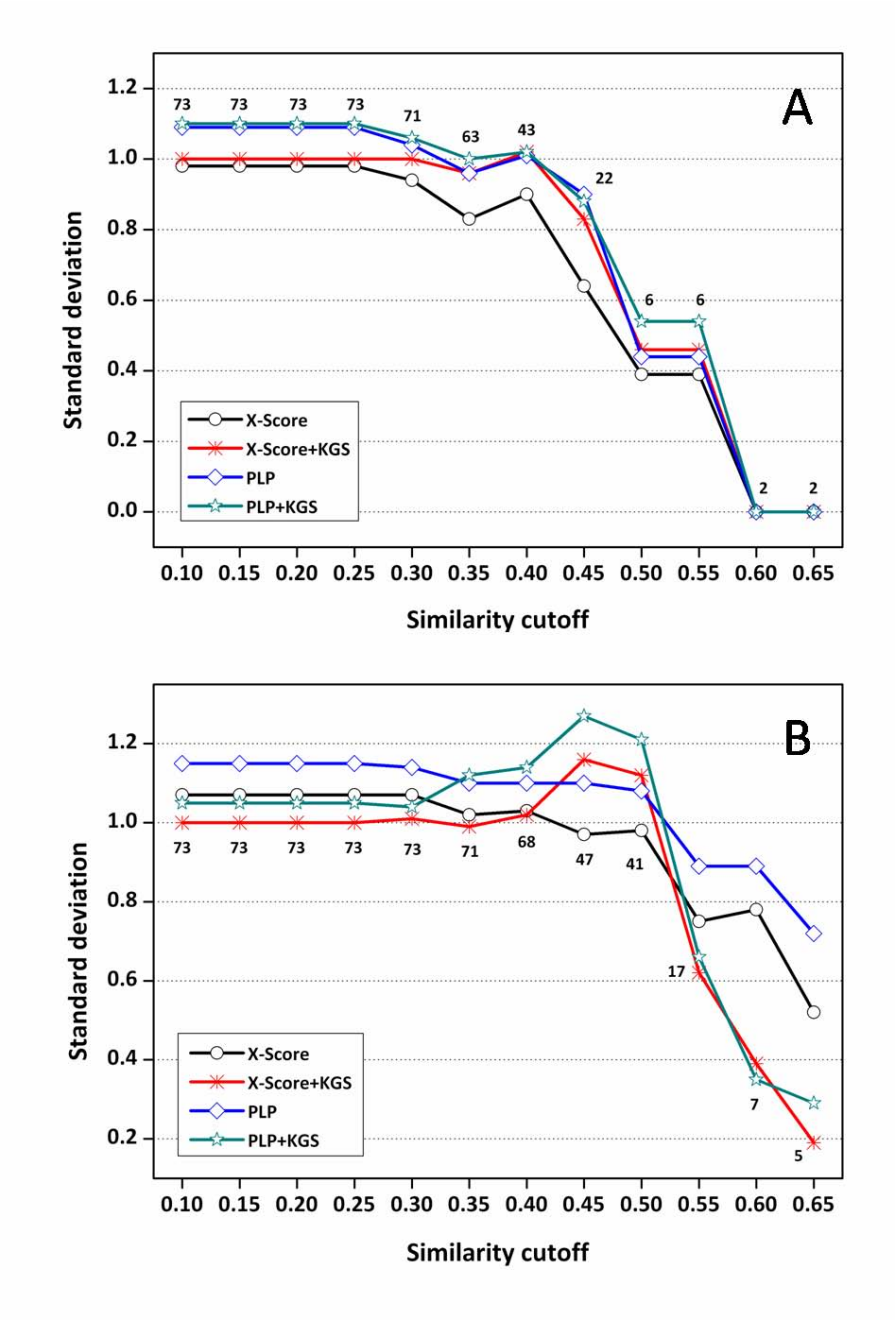
**Standard deviations (in log*K*_a _units) in fitting the experimentally measured binding constants of the trypsin complexes and the binding scores computed by X-Score and PLP in combination with the KGS strategy**. (*A*) Results obtained based on crystal structures (Set I). (*B*) Results obtained based on docking poses (Set II). The X axis indicates the similarity cutoffs used in defining reference complexes. The numbers indicated on this figure are the total numbers of the complexes considered at each similarity cutoff.

Compared to the performance on the HIV protease test set, X-Score and PLP are also more successful on this test set, producing standard deviations of 0.98 and 1.09 log units, respectively (Table 2). The standard deviations produced by X-Score, PLP and those in combination with the KGS strategy on this test set are plotted as a function of the similarity cutoffs used in reference searching in Figure 7. Detailed statistical results are summarized in the Additional file 1, Tables S5 and S6. Interestingly, when only the crystal structures are considered in scoring (Set I), both X-Score+KGS and PLP+KGS produce marginally larger standard deviations than X-Score and PLP alone on this test set (Figure 7A). When the docking poses are considered in scoring for instead (Set II), X-Score+KGS and PLP+KGS produce lower standard deviations than X-Score and PLP alone only when the similarity cutoff applied to defining reference complexes is really high (> 0.50) (Figure 7B). Unlike the results obtained on HIV protease complexes and carbonic anhydrase complexes, these results are quite unexpected, which drove us to look for the reason.

**Figure 8:**
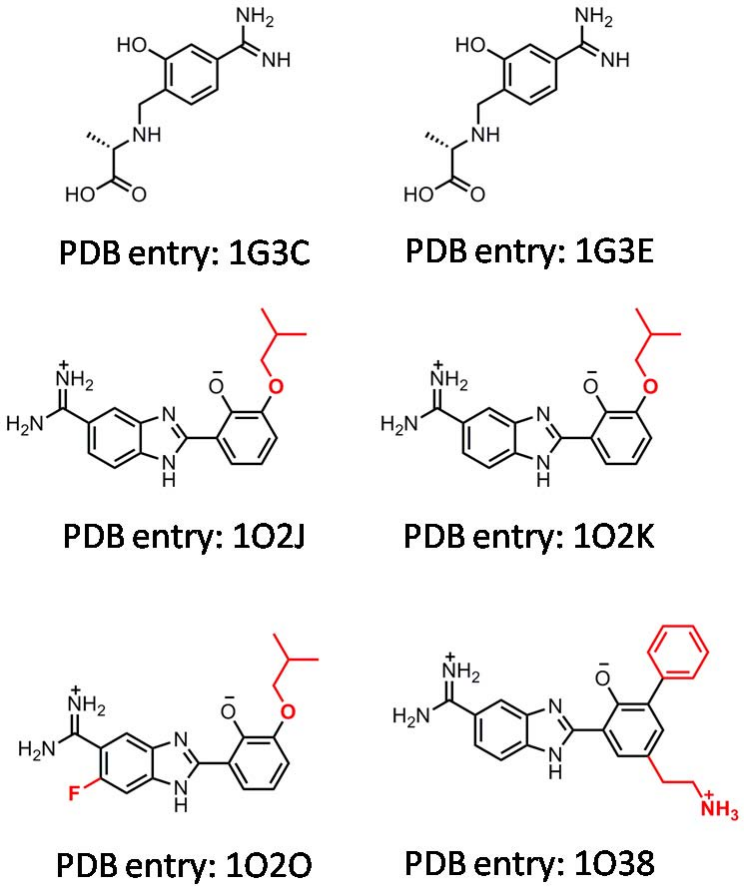
**Chemical structures of the six ligand molecules in the trypsin complexes listed in Table 4**.

The six pairs of trypsin complexes with the highest similarity in this test set are summarized in Table 4. The chemical structures of the six ligand molecules in the complexes are shown in Figure 8. One can see that in one case (PDB entry 1O2O) X-Score+KGS produced a notably larger error than X-Score alone. The experimental binding constants of complex 1O2O and its reference 1O2K (similarity = 0.58) are 6.36 and 6.92, respectively. The ligand molecule in complex 1O2O has one additional fluorine atom at the ortho-position of the aminidino group as compared to the ligand in complex 1O2K, which reduces the binding constant by approximately three folds. This reduction in binding affinity may be attributed to the unfavorable dipole-dipole repulsion between this fluorine atom and a nearby hydroxyl group on the side chain of Ser190 (F-O distance = 2.85 Å). Such dipole-dipole repulsions are indeed not taken into account by X-Score [35]. The binding scores produced by X-Score for complexes 1O2O and 1O2K are 6.27 and 6.08, respectively, giving a wrong ranking of these two complexes. Consequently, if X-Score+KGS is applied to predict the binding constant of 1O2O based on the known binding constant of 1O2K, it can only produce a binding score higher than the one of 1O2K, resulting in an even larger error (0.94 log units) than the one produced by X-Score alone (0.09 log units). We have observed that X-Score also produced wrong rankings of some other pairs of trypsin complexes, but 1O2O/1O2K is the one which contributes most to the larger deviations produced by X-Score+KGS on this test set.

**Table 4:** Information on the six pairs of trypsin complexes with the highest similarities

The query complex				The reference complex		
**PDB** **Code**	**-log*K*_*d*_(exp)**	**X-Score+** **KGS**	**X-Score**	**PDB** **Code**	**-log*K*_*d*_(exp)**	**Similarity**

1G3C	5.80	5.54	5.60	1G3E	5.38	0.67
1G3E	5.38	5.64	5.52	1G3C	5.80	0.67
1O2J	6.92	6.92	6.08	1O2K	6.92	0.58
1O2K	6.92	6.92	6.08	1O2J	6.92	0.58
1O2O	6.36	7.30	6.27	1O2K	6.92	0.58
1O38	6.82	6.48	6.33	1O2O	6.36	0.58

The above analysis suggests that failure of the KGS strategy in this case is caused directly by the intrinsic inaccuracy of the employed scoring function. In theory, our KGS strategy can effectively reduce the systematic errors in the predicted absolute binding constants. It thus becomes critical for the employed scoring function to produce correct rankings for the given complexes, a feature termed as "ranking power" in our recent study [22]. In that study, we demonstrated that today's best scoring functions are able to provide correct rankings for only 50-60% of the protein-ligand complex families under consideration. A much improved "ranking power" perhaps should be the primary aim for future scoring functions. Technically, developers of scoring functions may want to examine the ranking coefficient, such as the Spearman coefficient, more closely than the conventional Pearson coefficient between experimental binding constants and computed binding scores.

Another possible reason for the failure of the KGS strategy in this case lies in our algorithm for pharmacophore elucidation. In our study, a pharmacophore model actually represents a set of key protein-ligand interactions, and our KGS strategy relies on pharmacophore models for defining reference complexes. Such a pharmacophore model is dependent on the compositions as well as the conformations of both the protein and the ligand. Our algorithm only considers three types of features, i.e. hydrogen bond donor, hydrogen bond acceptor, and hydrophobic center. Other basic protein-ligand interactions, such as cation-pi interaction, are ignored for convenience. Besides, a pharmacophore has to contain a limited number of features to be practical. Therefore, some aspects in protein-ligand interactions may be missing. Simplification made in a pharmacophore model may lead to the choice of an inappropriate reference complex. Given these flaws, we have observed that our algorithm in some cases produced results controversial to commonsense. For example, complexes 1O2J and 1O2K are formed by an identical protein (trypsin) and an identical ligand (Figure 8), but the similarity between their pharmacophore models produced by our algorithm is only 0.58. Although these two complexes do exhibit some conformational difference in their binding pockets, one certainly expects a higher similarity score in this case. Our algorithm for pharmacophore elucidation certainly can be improved further.

### Comparison with other targeted scoring methods

As mentioned in the *Introduction *section, current targeted scoring functions [55-58] are developed typically through re-parameterization or modification on existing all-purpose scoring functions, which often relies on some sophisticated statistical procedures. In contrast, our KGS strategy works in a different fashion. There are two basic modules in the framework of KGS: one is a scoring method, and the other is a method for defining the reference complexes. As for the scoring method, we have tested X-Score and PLP in this study. But it can be any well-validated scoring function or other approach. Once the scoring method is chosen, it can be applied as is without re-parameterization or modification. This is important since many programs are available to the end-users as black boxes. As for the method for defining reference complexes, our current algorithm is based on comparison of structure-based pharmacophore models of relevant protein-ligand complexes. Other algorithms of course may be considered as well, such as the protein-ligand interaction fingerprints [71,72]. Thus, these two modules can be chosen independently, and it is in principle flexible to combine them. This is a notable technical advantage of our KGS strategy, and that is why we refer to it as a strategy rather than a particular method.

Compared to most targeted scoring functions, another technical advantage of our KGS strategy is that its application is in principle not limited to certain classes of targets. In order to apply the KGS strategy, an external database of protein-ligand complexes with known three-dimensional structures and experimental binding data needs to be supplied as a knowledge set. If one needs to consider protein-ligand complexes of various types, this knowledge set may consist of a sufficient number of protein-ligand complexes of various types, such as the PDBbind database. Otherwise, if one's study focuses on a particular class of protein-ligand complexes, one may want to supply a knowledge set only consisting of relevant protein-ligand complexes, such as the test sets used in this study. In practice, most researchers study a certain congeneric class of ligands bound to a common target protein, and thus the latter approach is more suitable for this purpose. Note that in such a case, one can even employ a targeted scoring function, if available, as the internal scoring method for the KGS strategy to obtain more accurate results. In this sense, our KGS strategy is fully compatible with targeted scoring functions.

It should be mentioned that our KGS strategy is similar to the AutoShim method proposed by Martin and Sullivan [73] in certain aspects. AutoShim does not require re-parameterization of scoring function either, and in principle does not tie to any specific scoring functions. According to AutoShim, point-pharmacophore like "shims" are generated in the binding pocket on the target protein. These "shims" are then weighted by partial least squares (PLS) regression to adjust the outcomes of the Flo+ scoring function in order to better reproduce known binding data. Nevertheless, AutoShim is basically a 3D-QSAR model, which integrates the Flo+ score as well as several hundred of descriptors ("shims"). Such a model normally needs to be carefully validated to avoid over-fitting since it relies on so many parameters. As a matter of fact, Martin et al mentioned that their PLS model produced comparable results with or without the Flo+ score in the entire descriptor set (*R*^2 ^= 0.60 vs. *R*^2 ^= 0.56). In contrast, the internal scoring function (e.g. X-Score or PLP) in our KGS strategy plays an indispensable role. Another common aspect between AutoShim and our KGS strategy is that both methods require a training set of protein-ligand complexes with known experimental binding data. As a QSAR-like method, AutoShim relies much on this training set for model calibration. For our KGS strategy, the training set is also used for deriving the scaling parameter *k *in Equation 4, but it serves primarily as a knowledge set for defining appropriate reference complexes. Using an appropriate reference contributes most to the improved accuracy of our KGS strategy in binding affinity prediction.

Finally, as mentioned repeatedly in this article, our KGS strategy computes the binding constant of a given protein-ligand complex based on the known binding constant of a reference complex. It is essentially an interpolation method. We all know that more accurate results can be obtained through interpolation if more known data exist in the problem space. The knowledge of protein-ligand binding data is certainly in a constant increase. For example, binding data included in the PDBbind database increase by approximately 25% each year. Thus, application of the KGS strategy will hopefully produce more and more accurate results with the increase in such knowledge. In contrast, other targeted scoring functions benefit indirectly from the increasing knowledge of binding data since re-calibration on a larger data set does not necessarily produce a more accurate model. Besides, the majority of experimental binding data as well as crystal structures are actually owned by pharmaceutical companies, which are not available to the public for understandable reasons. Researchers from pharmaceutical companies can supply their in-house data as the external knowledge set required by the KGS strategy so that their own data can be effectively utilized in binding affinity prediction as well.

## Conclusions

We have developed a general strategy, i.e. knowledge-guided scoring (KGS), for improving the accuracy of scoring functions in binding affinity prediction. Our KGS strategy computes the binding constant of a given protein-ligand complex based on the known binding constant of an appropriate reference complex. The reference complex is required to share a similar pattern of protein-ligand interactions to that of the complex of interest. Thus, some uncertain factors in protein-ligand binding, which are difficult to be accurately considered by scoring functions, may cancel out in computation, resulting in more accurate prediction of absolute binding affinities.

Our KGS strategy was evaluated in combination with X-Score and PLP on three sets of protein-ligand complexes. As for the HIV protease complexes, X-Score and PLP alone failed to provide acceptable prediction of binding constants; while both X-Score+KGS and PLP+KGS demonstrated notably improved performance especially when the similarity cutoff used in reference searching was relatively high. As for the carbonic anhydrase complexes and trypsin complexes, both X-Score and PLP were able to provide reasonable results by themselves. Application of the KGS strategy in these two cases only produced marginally better or comparable results due to limited remaining room for improvement. An interesting observation is that X-Score+KGS and PLP+KGS produced converged results despite the difference in the outcomes of X-Score and PLP alone. This prompts that application of the KGS strategy may save the end-users, at least to some extents, from the troublesome evaluation and shopping among different scoring functions. Besides, the standard deviations between the experimental and computed binding constants produced by X-Score+KGS and PLP+KGS are below 1.0 log*K*_*d *_units (corresponding to 1.36 kcal/mol in binding free energy at room temperature) on all three test sets when the similarity cutoff is over 0.40. This level of accuracy is certainly acceptable for high-throughput tasks in structure-based drug design. It however remains to be verified if application of KGS is able to achieve this level accuracy consistently on other classes of protein-ligand complexes.

In principle, KGS can be applied in combination with any existing scoring functions. Unlike many other targeted scoring functions, KGS does not require re-parameterization of the given scoring function, and its application is not limited to certain classes of protein-ligand complexes. In addition, KGS is essentially an interpolation method, and thus its effectiveness is in theory proportional to the ever-increasing knowledge of experimental binding data. In-house collections of binding data can be effectively utilized by KGS in computation as well. Compared to other targeted scoring functions, these features make our KGS strategy a more practical remedy for current scoring functions to improve their accuracy in binding affinity prediction. Nevertheless, we did not attempt to test the KGS strategy in this study to see if it also helps with binding mode prediction, or achieves higher success rates in cross-docking or hit rates in virtual screening. For those purposes, we believe that our current algorithms for applying KGS may need certain adjustments.

## Authors' contributions

TC implemented the algorithms for pharmacophore elucidation and comparison, evaluated the KGS strategy, performed subsequent statistical analyses, and drafted this article. ZL prepared the three test sets used in the evaluation of the KGS strategy and contributed to programming as well. RW conceived of this study, coordinated and supervised its execution, revised the manuscript substantially for important intellectual contents. All authors have read and approved the final manuscript.

## Supplementary Material

Additional file 1 **Supplementary tables S1-S6**. Detailed statistical results for making Figures 4, 6 and 7, which were produced by X-Score and PLP in combination with the KGS strategy on three test sets based on native binding poses as well as computer-generated docking poses. • Table S1: Statistical results obtained on the HIV protease complexes using native binding poses in scoring • Table S2: Statistical results obtained on the HIV protease complexes using docking poses in scoring • Table S3: Statistical results obtained on the carbonic anhydrase complexes using native binding poses in scoring • Table S4: Statistical results obtained on the carbonic anhydrase complexes using docking poses in scoring • Table S5: Statistical results obtained on the trypsin complexes using native binding poses in scoring • Table S6: Statistical results obtained on the trypsin complexes using docking poses in scoring Click here for file
