# Supplementary material for: A knowledge-guided strategy for improving the accuracy of scoring functions in binding affinity prediction
Source: BMC Bioinformatics. 2010 Apr 17;11:193. doi: 10.1186/1471-2105-11-193 (PMC2868011; doi:10.1186/1471-2105-11-193)
Supplement: Additional file 1 — Supplementary tables S1-S6. Detailed statistical results for making Figures 4, 6 and 7, which were produced by X-Score and PLP in combination with the KGS strategy on three test sets based on native binding poses as well as computer-generated docking poses. • Table S1: Statistical results obtained on the HIV protease complexes using native binding poses in scoring • Table S2: Statistical results obtained on the HIV protease complexes using docking poses in scoring • Table S3: Statistical results obtained on the carbonic anhydrase complexes using native binding poses in scoring • Table S4: Statistical results obtained on the carbonic anhydrase complexes using docking poses in scoring • Table S5: Statistical results obtained on the trypsin complexes using native binding poses in scoring • Table S6: Statistical results obtained on the trypsin complexes using docking poses in scoring [file 1471-2105-11-193-S1.DOC]

**Additional File 1**

**A knowledge-guided strategy for improving the accuracy of scoring functions in binding affinity prediction**

Tiejun Cheng, Zhihai Liu, and Renxiao Wang*

*State Key Laboratory of Bioorganic Chemistry, Shanghai Institute of Organic Chemistry, Chinese Academy of Sciences, 345 Lingling Road, Shanghai 200032, People’s Republic of China*

*Contact e-mail: wangrx@mail.sioc.ac.cn*

**Table S1.** Statistical results obtained on the HIV protease complexes using native binding poses in scoring

|  |  | **X-Score + KGS** | | **X-Score** | | **PLP + KGS** | | **PLP** | |
| --- | --- | --- | --- | --- | --- | --- | --- | --- | --- |
| **Similarity**  **Cutoff** | ***N ^a^*** | ***R*** | ***SD*** | ***R*** | ***SD*** | ***R*** | ***SD*** | ***R*** | ***SD*** |
| 0.10 | 112 | 0.388 | 1.51 | 0.329 | 1.55 | 0.346 | 1.54 | 0.190 | 1.61 |
| 0.15 | 112 | 0.388 | 1.51 | 0.329 | 1.55 | 0.346 | 1.54 | 0.190 | 1.61 |
| 0.20 | 111 | 0.352 | 1.50 | 0.291 | 1.54 | 0.302 | 1.53 | 0.136 | 1.59 |
| 0.25 | 109 | 0.320 | 1.49 | 0.227 | 1.53 | 0.270 | 1.51 | 0.069 | 1.56 |
| 0.30 | 109 | 0.320 | 1.49 | 0.227 | 1.53 | 0.270 | 1.51 | 0.069 | 1.56 |
| 0.35 | 89 | 0.380 | 1.48 | 0.215 | 1.57 | 0.335 | 1.51 | 0.009 | 1.60 |
| 0.40 | 44 | 0.689 | 1.10 | 0.371 | 1.41 | 0.653 | 1.15 | 0.046 | 1.51 |
| 0.45 | 19 | 0.832 | 0.71 | 0.357 | 1.20 | 0.843 | 0.69 | 0.460 | 1.14 |
| 0.50 | 8 | 0.891 | 0.62 | 0.040 | 1.36 | 0.911 | 0.56 | 0.412 | 1.24 |
| 0.55 | 8 | 0.891 | 0.62 | 0.040 | 1.36 | 0.911 | 0.56 | 0.412 | 1.24 |
| 0.60 | 5 | 0.856 | 0.84 | 0.518 | 1.38 | 0.878 | 0.78 | 0.914 | 0.66 |
| 0.65 | 5 | 0.856 | 0.84 | 0.518 | 1.38 | 0.878 | 0.78 | 0.914 | 0.66 |

*^a^* Number of the complexes considered in correlation analysis. An appropriate reference complex was found for each of them at the given similarity cutoff.

**Table S2.** Statistical results obtained on the HIV protease complexes using docking poses in scoring

|  |  | **X-Score + KGS** | | **X-Score** | | **PLP + KGS** | | **PLP** | |
| --- | --- | --- | --- | --- | --- | --- | --- | --- | --- |
| **Similarity**  **Cutoff** | ***N ^a^*** | ***R*** | ***SD*** | ***R*** | ***SD*** | ***R*** | ***SD*** | ***R*** | ***SD*** |
| 0.10 | 112 | 0.215 | 1.60 | 0.316 | 1.55 | 0.089 | 1.63 | 0.157 | 1.62 |
| 0.15 | 112 | 0.215 | 1.60 | 0.316 | 1.55 | 0.089 | 1.63 | 0.157 | 1.62 |
| 0.20 | 112 | 0.215 | 1.60 | 0.316 | 1.55 | 0.089 | 1.63 | 0.157 | 1.62 |
| 0.25 | 112 | 0.215 | 1.60 | 0.318 | 1.55 | 0.089 | 1.63 | 0.161 | 1.62 |
| 0.30 | 111 | 0.174 | 1.58 | 0.278 | 1.54 | 0.033 | 1.61 | 0.105 | 1.60 |
| 0.35 | 110 | 0.177 | 1.59 | 0.264 | 1.56 | 0.077 | 1.61 | 0.077 | 1.61 |
| 0.40 | 104 | 0.349 | 1.51 | 0.282 | 1.55 | 0.281 | 1.55 | 0.095 | 1.61 |
| 0.45 | 59 | 0.569 | 1.29 | 0.436 | 1.41 | 0.555 | 1.30 | 0.359 | 1.46 |
| 0.50 | 59 | 0.569 | 1.29 | 0.436 | 1.41 | 0.555 | 1.30 | 0.359 | 1.46 |
| 0.55 | 18 | 0.705 | 0.88 | 0.289 | 1.18 | 0.731 | 0.84 | 0.487 | 1.08 |
| 0.60 | 7 | 0.815 | 0.81 | 0.500 | 1.22 | 0.842 | 0.76 | 0.839 | 0.77 |
| 0.65 | 7 | 0.815 | 0.81 | 0.500 | 1.22 | 0.842 | 0.76 | 0.839 | 0.77 |

*^a^* Number of the complexes considered in correlation analysis. An appropriate reference complex was found for each of them at the given similarity cutoff.

**Table S3. Statistical results obtained on the carbonic anhydrase complexes using native binding poses in scoring**

|  |  | **X-Score + KGS** | | **X-Score** | | **PLP + KGS** | | **PLP** | |
| --- | --- | --- | --- | --- | --- | --- | --- | --- | --- |
| **Similarity**  **Cutoff** | ***N ^a^*** | ***R_p_*** | ***SD*** | ***R_p_*** | ***SD*** | ***R_p_*** | ***SD*** | ***R_p_*** | ***SD*** |
| 0.10 | 44 | 0.730 | 0.95 | 0.648 | 1.06 | 0.762 | 0.90 | 0.690 | 1.01 |
| 0.15 | 44 | 0.730 | 0.95 | 0.648 | 1.06 | 0.762 | 0.90 | 0.690 | 1.01 |
| 0.20 | 44 | 0.730 | 0.95 | 0.648 | 1.06 | 0.762 | 0.90 | 0.690 | 1.01 |
| 0.25 | 43 | 0.690 | 0.91 | 0.556 | 1.04 | 0.720 | 0.87 | 0.603 | 1.00 |
| 0.30 | 40 | 0.636 | 0.75 | 0.195 | 0.95 | 0.665 | 0.72 | 0.277 | 0.93 |
| 0.35 | 37 | 0.754 | 0.64 | 0.248 | 0.94 | 0.720 | 0.67 | 0.298 | 0.93 |
| 0.40 | 27 | 0.589 | 0.58 | 0.603 | 0.57 | 0.694 | 0.52 | 0.482 | 0.63 |
| 0.45 | 13 | 0.830 | 0.46 | 0.835 | 0.45 | 0.772 | 0.52 | 0.171 | 0.81 |
| 0.50 | 6 | 0.648 | 0.40 | 0.260 | 0.51 | 0.552 | 0.44 | 0.288 | 0.51 |
| 0.55 | 5 | 0.655 | 0.46 | 0.233 | 0.59 | 0.949 | 0.19 | 0.852 | 0.32 |
| 0.60 | 2 | 1.000 | 0.00 | 1.000 | 0.00 | 1.000 | 0.00 | 1.000 | 0.00 |

*^a^* Number of the complexes considered in correlation analysis. An appropriate reference complex was found for each of them at the given similarity cutoff.

**Table S4. Statistical results obtained on the carbonic anhydrase complexes using docking poses in scoring**

|  |  | **X-Score + KGS** | | **X-Score** | | **PLP + KGS** | | **PLP** | |
| --- | --- | --- | --- | --- | --- | --- | --- | --- | --- |
| **Similarity**  **Cutoff** | ***N ^a^*** | ***R_p_*** | ***SD*** | ***R_p_*** | ***SD*** | ***R_p_*** | ***SD*** | ***R_p_*** | ***SD*** |
| 0.10 | 44 | 0.512 | 1.20 | 0.617 | 1.09 | 0.628 | 1.08 | 0.675 | 1.03 |
| 0.15 | 44 | 0.512 | 1.20 | 0.617 | 1.09 | 0.628 | 1.08 | 0.675 | 1.03 |
| 0.20 | 44 | 0.512 | 1.20 | 0.617 | 1.09 | 0.628 | 1.08 | 0.675 | 1.03 |
| 0.25 | 44 | 0.511 | 1.20 | 0.623 | 1.09 | 0.649 | 1.06 | 0.689 | 1.01 |
| 0.30 | 43 | 0.531 | 1.18 | 0.615 | 1.10 | 0.671 | 1.03 | 0.699 | 1.00 |
| 0.35 | 40 | 0.181 | 0.95 | 0.094 | 0.97 | 0.332 | 0.92 | 0.330 | 0.92 |
| 0.40 | 39 | 0.480 | 0.86 | 0.141 | 0.97 | 0.546 | 0.82 | 0.434 | 0.89 |
| 0.45 | 28 | 0.700 | 0.58 | 0.626 | 0.64 | 0.768 | 0.52 | 0.657 | 0.62 |
| 0.50 | 25 | 0.681 | 0.63 | 0.598 | 0.69 | 0.733 | 0.58 | 0.578 | 0.70 |
| 0.55 | 16 | 0.607 | 0.58 | 0.685 | 0.53 | 0.488 | 0.64 | 0.079 | 0.73 |
| 0.60 | 12 | 0.800 | 0.45 | 0.634 | 0.57 | 0.670 | 0.55 | 0.203 | 0.73 |
| 0.65 | 4 | 0.945 | 0.17 | 0.955 | 0.15 | 0.477 | 0.44 | 0.518 | 0.43 |
| 0.70 | 3 | 0.735 | 0.15 | 0.334 | 0.20 | 0.961 | 0.06 | 0.856 | 0.11 |

*^a^* Number of the complexes considered in correlation analysis. An appropriate reference complex was found for each of them at the given similarity cutoff.

**Table S5. Statistical results obtained on the trypsin complexes using native binding poses in scoring**

|  |  | **X-Score + KGS** | | **X-Score** | | **PLP + KGS** | | **PLP** | |
| --- | --- | --- | --- | --- | --- | --- | --- | --- | --- |
| **Similarity**  **Cutoff** | ***N ^a^*** | ***R_p_*** | ***SD*** | ***R_p_*** | ***SD*** | ***R_p_*** | ***SD*** | ***R_p_*** | ***SD*** |
| 0.10 | 73 | 0.805 | 1.00 | 0.815 | 0.98 | 0.761 | 1.10 | 0.762 | 1.09 |
| 0.15 | 73 | 0.805 | 1.00 | 0.815 | 0.98 | 0.761 | 1.10 | 0.762 | 1.09 |
| 0.20 | 73 | 0.805 | 1.00 | 0.815 | 0.98 | 0.761 | 1.10 | 0.762 | 1.09 |
| 0.25 | 73 | 0.805 | 1.00 | 0.815 | 0.98 | 0.761 | 1.10 | 0.762 | 1.09 |
| 0.30 | 71 | 0.793 | 1.00 | 0.820 | 0.94 | 0.761 | 1.06 | 0.776 | 1.04 |
| 0.35 | 63 | 0.811 | 0.96 | 0.863 | 0.83 | 0.794 | 1.00 | 0.811 | 0.96 |
| 0.40 | 43 | 0.826 | 1.02 | 0.868 | 0.90 | 0.828 | 1.02 | 0.830 | 1.01 |
| 0.45 | 22 | 0.824 | 0.83 | 0.899 | 0.64 | 0.796 | 0.88 | 0.790 | 0.90 |
| 0.50 | 6 | 0.779 | 0.46 | 0.844 | 0.39 | 0.675 | 0.54 | 0.800 | 0.44 |
| 0.55 | 6 | 0.779 | 0.46 | 0.844 | 0.39 | 0.675 | 0.54 | 0.800 | 0.44 |
| 0.60 | 2 | 1.000 | 0.00 | 1.000 | 0.00 | 1.000 | 0.00 | 1.000 | 0.00 |
| 0.65 | 2 | 1.000 | 0.00 | 1.000 | 0.00 | 1.000 | 0.00 | 1.000 | 0.00 |

*^a^* Number of the complexes considered in correlation analysis. An appropriate reference complex was found for each of them at the given similarity cutoff.

**Table S6. Statistical results obtained on the trypsin complexes using docking poses in scoring**

|  |  | **X-Score + KGS** | | **X-Score** | | **PLP + KGS** | | **PLP** | |
| --- | --- | --- | --- | --- | --- | --- | --- | --- | --- |
| **Similarity**  **Cutoff** | ***N ^a^*** | ***R_p_*** | ***SD*** | ***R_p_*** | ***SD*** | ***R_p_*** | ***SD*** | ***R_p_*** | ***SD*** |
| 0.10 | 73 | 0.806 | 1.00 | 0.776 | 1.07 | 0.785 | 1.05 | 0.735 | 1.15 |
| 0.15 | 73 | 0.806 | 1.00 | 0.776 | 1.07 | 0.785 | 1.05 | 0.735 | 1.15 |
| 0.20 | 73 | 0.806 | 1.00 | 0.776 | 1.07 | 0.785 | 1.05 | 0.735 | 1.15 |
| 0.25 | 73 | 0.806 | 1.00 | 0.776 | 1.07 | 0.785 | 1.05 | 0.735 | 1.15 |
| 0.30 | 73 | 0.802 | 1.01 | 0.776 | 1.07 | 0.789 | 1.04 | 0.737 | 1.14 |
| 0.35 | 71 | 0.799 | 0.99 | 0.786 | 1.02 | 0.735 | 1.12 | 0.747 | 1.10 |
| 0.40 | 68 | 0.797 | 1.02 | 0.793 | 1.03 | 0.740 | 1.14 | 0.756 | 1.10 |
| 0.45 | 47 | 0.735 | 1.16 | 0.823 | 0.97 | 0.667 | 1.27 | 0.765 | 1.10 |
| 0.50 | 41 | 0.776 | 1.12 | 0.836 | 0.98 | 0.731 | 1.21 | 0.793 | 1.08 |
| 0.55 | 17 | 0.927 | 0.62 | 0.892 | 0.75 | 0.919 | 0.66 | 0.846 | 0.89 |
| 0.60 | 7 | 0.954 | 0.39 | 0.793 | 0.78 | 0.962 | 0.35 | 0.723 | 0.89 |
| 0.65 | 5 | 0.979 | 0.19 | 0.834 | 0.52 | 0.951 | 0.29 | 0.630 | 0.72 |

*^a^* Number of the complexes considered in correlation analysis. An appropriate reference complex was found for each of them at the given similarity cutoff.
